# Bibliometric Analysis of Research Trends Related to the Publication of Clinical Trials on the Treatment of Temporomandibular Disorders Between 1973 and 2023

**DOI:** 10.1155/ijod/8594730

**Published:** 2025-08-06

**Authors:** Yens Mendoza-Martiarena, Miguel Ángel Norabuena-Robles, Kilder Maynor Carranza-Samanez, Claudia Denisse Piscoche-Rodríguez

**Affiliations:** ^1^Research Group in Dental Sciences, School of Dentistry, Universidad Científica del Sur, Lima, Peru; ^2^School of Medical Technology, School of Medicine, Universidad Nacional Mayor de San Marcos, Lima, Peru; ^3^Academic Department, School of Dentistry, Universidad Nacional Mayor de San Marcos, Lima, Peru

**Keywords:** clinical trial, orofacial pain, temporomandibular disorder, temporomandibular joint

## Abstract

**Objective:** To perform a bibliometric analysis of clinical trials (CTs) that evaluated the effectiveness of treatments for temporomandibular disorders (TMDs) between 1973 and 2023.

**Materials and methods:** This bibliometric study analyzed CT on TMD treatment identified in Scopus and Web of Science (WoS) databases through a query in MeSH terms. The main characteristics analyzed were year of publication, institutions, authors, citations, and keywords. Data were retrieved on March 18, 2024. VOSviewer and CiteSpace tools were used to create bibliometric networks and other visualizations.

**Results:** A total of 559 CTs evaluating TMD treatments were identified. A remarkable growth was observed in the last 10 years, with USA, Brazil, and Turkey being the main contributors in publications. Conservative therapies (COTs) such as physiotherapy and the use of occlusal splints, together with minimally invasive therapies (MITs), such as joint level injections, were the most applied in recent years. The Journal of Oral and Maxillofacial Surgery and Cranio—Journal of Craniomandibular and Sleep Practice were the leading journals in the field and with an important number of citations. The University of São Paulo and Universidade Nove de Julho were the institutions with the highest contribution of CTs. The most prolific author had 12 publications.

**Conclusion:** Clinical research on TMD treatments is extensive and growing; however, there are large differences in the number of CTs related to emerging therapies. Future studies should focus on the implementation of high-quality CTs by fostering international cooperations and expanding knowledge on emerging COTs and MITs.

## 1. Introduction

Temporomandibular disorders (TMDs) are a group of musculoskeletal pathologies that affect the world population, with a prevalence that varies between 26% and 47% [1]. Common signs and symptoms presented in TMDs include muscle and orofacial pain, limitation of masticatory function, alteration of the structures surrounding the temporomandibular joint (TMJ), orofacial pain, and psychosocial alterations [2, 3]. The treatment strategies available are varied and depend on the cause, severity, and location of the pain, among others [4, 5]. For many years, the effectiveness of treatments for TMDs has been studied, with the results depending on the physical or psychosocial basis associated with these disorders, thereby making the approach to TMD complex in dentistry, physiotherapy, psychology, and medicine [4]. The complexity and confluence of symptoms and dimensions of health that TMDs affect [6, 7] has led to variable knowledge related to TMD therapies analyzed in clinical trials (CTs) [6].

CTs have rigorous research designs that provide the most evidence and allow testing the safety, efficacy, and/or effectiveness of treatments in people with a particular health condition [8]. Since the identification and classification of the Costen syndrome [9], a number of CTs have sought to evaluate the effectiveness of different treatments, ranging from physiotherapy [7], drug- [10] or toxin-based therapy, intra-articular injections [4, 11], alloplastic TMJ surgery [12, 13], and combination therapies, among others. CTs on TMDs have expanded throughout the world, but only a few countries and researchers are considered as authorities in the treatment for TMDs because of their level of research contribution. Due to the variability of TMD therapies and the continued interest in offering evidence-based treatments with the design of CTs, it is necessary to determine the trends and direction of research within the TMD treatment pipeline through bibliometrics.

Bibliometric techniques are quantitative strategies for tracking research over time, understanding its evolution and identifying new knowledge [14]. This allows a glimpse of the next direction that a line of research will take for evidence-based decision making for the implementation of health policies. Thanks to their flexibility, CTs with multiple approaches, including trials aimed at the prevention, evaluation, diagnosis, and treatment of TMDs, can be developed due to their methodological adaptability [8, 15]. Currently, there are very few bibliometric studies focused on evaluating research on treatments for TMD in CTs. Therefore, our objective was to determine the trends, productivity indicators, and collaborations related to CT conducted to test the efficacy of treatments in TMD.

## 2. Methods and Materials

### 2.1. Study Design

This bibliometric study analyzed all the CTs that evaluated the effectiveness of the treatments applied in people with TMD. Data extraction was performed on May 18, 2024, and the Scopus and Web of Science (WoS) Core Collection databases were used. All records found up to 2023 were retrieved considering the eligibility criteria without the need to perform a sample calculation.

### 2.2. Search Strategy

MeSH terms were used for keyword selection. For the search of the articles, words such as: clinical trial, randomized trial, Controlled Clinical Trial, TMJ, TMJ disorder were used, considering other synonymous terms found in the Medical Subject Headings (MeSH) and were joined with the Boolean operator “OR” and “AND.” This strategy was applied for both Scopus and WoS. In the latter, the Science Citation Index Expanded (SCI-EXPANDED) was selected as the edition. The search strategy was as follows:

(((TI = (“Temporomandibular Joint” OR “Joint, Temporomandibular” OR “Joints, Temporomandibular” OR “Temporomandibular Joints” OR “TMJ” OR “Disorder, Temporomandibular Joint” OR “Disorders, Temporomandibular Joint” OR “Joint Disorder, Temporomandibular” OR “Joint Disorders, Temporomandibular” OR “Temporomandibular Joint Disorder” OR “TMJ Disorders” OR “Disorder, TMJ” OR “Disorders, TMJ” OR “TMJ Disorder” OR “Temporomandibular Disorders” OR “Disorder, Temporomandibular” OR “Disorders, Temporomandibular” OR “Temporomandibular Disorder” OR “Temporomandibular Joint Diseases” OR “Disease, Temporomandibular Joint” OR “Diseases, Temporomandibular Joint” OR “Joint Disease, Temporomandibular” OR “Joint Diseases, Temporomandibular” OR “Temporomandibular Joint Disease” OR “TMJ Diseases” OR “Disease, TMJ” OR “Diseases, TMJ” OR “TMJ Disease” OR “Temporomandibular Joint Disorders” OR “Temporomandibular Joint Dysfunction Syndrome” OR “Myofascial Pain Dysfunction Syndrome, Temporomandibular Joint” OR “TMJ Syndrome” OR “Syndrome, TMJ” OR “Costen's Syndrome” OR “Costen Syndrome” OR “Costens Syndrome” OR “Syndrome, Costen's” OR “Temporomandibular Joint Syndrome” OR “Joint Syndrome, Temporomandibular” OR “Syndrome, Temporomandibular Joint” OR “Temporomandibular Joint Disorders” OR “Temporomandibular Joint Disorder” OR “Disorder, Temporomandibular Joint” OR “Disorders, Temporomandibular Joint” OR “Joint Disorder, Temporomandibular” OR “Joint Disorders, Temporomandibular” OR “Temporomandibular Joint Disorder” OR “TMJ Disorders” OR “Disorder, TMJ” OR “Disorders, TMJ” OR “TMJ Disorder” OR “Temporomandibular Disorders” OR “Disorder, Temporomandibular” OR “Disorders, Temporomandibular” OR “Temporomandibular Disorder” OR “Temporomandibular Joint Diseases” OR “Disease, Temporomandibular Joint” OR “Diseases, Temporomandibular Joint” OR “Joint Disease, Temporomandibular” OR “Joint Diseases, Temporomandibular” OR “Temporomandibular Joint Disease” OR “TMJ Diseases” OR “Disease, TMJ” OR “Diseases, TMJ” OR “TMJ Disease")) AND (TI = (“Randomized clinical trial” OR “clinical trial” OR “randomized trial” OR “Controlled Clinical Trial” OR “Pragmatic Clinical Trial” OR “randomized controlled trial” OR “randomized control trial” OR “control trial” OR “controlled trial” OR “experimental stud*⁣*^*∗*^”) OR AB = (“Randomized clinical trial” OR “clinical trial” OR “randomized trial” OR “Controlled Clinical Trial” OR “Pragmatic Clinical Trial” OR “randomized controlled trial” OR “randomized control trial” OR “control trial” OR “controlled trial” OR “experimental stud*⁣*^*∗*^”) OR KP = (“Randomized clinical trial” OR “clinical trial” OR “randomized trial” OR “Controlled Clinical Trial” OR “Pragmatic Clinical Trial” OR “randomized controlled trial” OR “randomized control trial” OR “control trial” OR “controlled trial” OR “experimental stud*⁣*^*∗*^”) OR AK = (“Randomized clinical trial” OR “clinical trial” OR “randomized trial” OR “Controlled Clinical Trial” OR “Pragmatic Clinical Trial” OR “randomized controlled trial” OR “randomized control trial” OR “control trial” OR “controlled trial” OR “experimental stud*⁣*^*∗*^”))))

**Figure 1:**
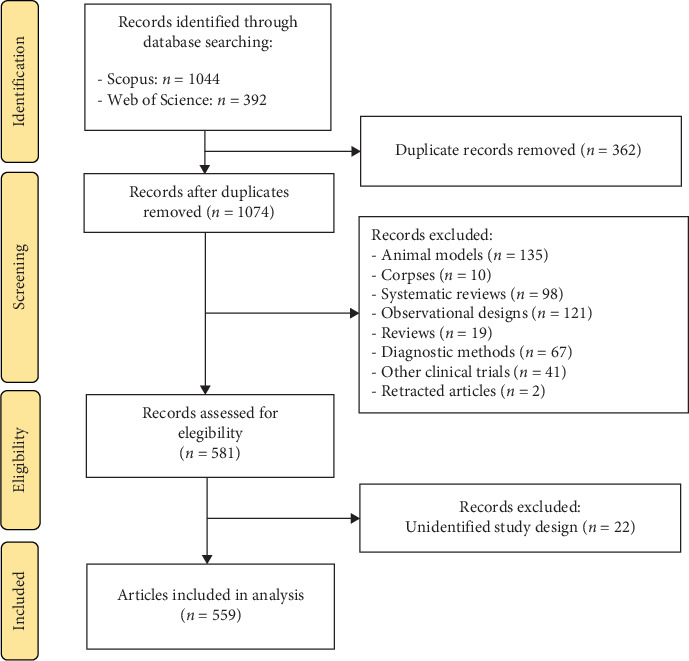
Flowchart of study selection.

The search and selection of research was done until the year 2023 according to the eligibility criteria. Only CTs in humans, CTs that evaluated treatments for TMD, controlled and uncontrolled, randomized, and nonrandomized RCTs were included. Animal or cadaver CTs and human CTs that evaluated diagnostic tests or preventive practices for TMD were excluded. There were no filters on the type of language. The records obtained with the search strategy of both databases were exported to a reference manager (Mendeley) for the elimination of duplicates. Record verification was done by two persons (YVMM and CDPR); YVMM performed the search and first screening and CDPR validated the records by a second screening ensuring that only CTs were included in the analysis. The level of agreement among the reviewers was evaluated using the Kappa coefficient (*k* = 0.92). Los procedimientos de limpieza comprendieron la normalización de nombres *y* apellidos de autor, de instituciones *y* de revistas. Este procedimiento se hizo de forma manual. The selection and exclusion of records can be seen in more detail in Figure 1.

### 2.3. Data Analysis

The VOSviewer tool [16] 1.6.23 was used to construct the bibliometric networks. The networks allowed representing the main authors, countries, and institutions through co-authorship analysis with minimum thresholds of 2, 5, and 3, respectively. The analysis of co-citation of journals and authors was performed with a minimum number of citations of 13 for both networks and co-occurrence analysis considering a full counting with a minimum co-occurrence threshold of 29. All networks were constructed using the optimized modularity clustering algorithm [16], based on a variant of Louvain's method. The size of the network nodes represented the number of times an element appeared, the thickness of the lines determined the strength of co-occurrence within the network. The networks were grouped by clusters, where the same color represented their thematic similarity. In addition, trends of the most applied TMD treatments were determined. CiteSpace 6.6.R1 [17] was used to perform keyword and reference analysis using bursts. Burst analyses were performed by applying the Kleinberg algorithm [17]. The analysis period was from 2014 to 2023 with annual intervals (slice length = 1). Keywords and references were selected based on their burst strength considering a minimum duration of 1 year.

## 3. Results

### 3.1. Analysis of General Research Trends

**Figure 2:**
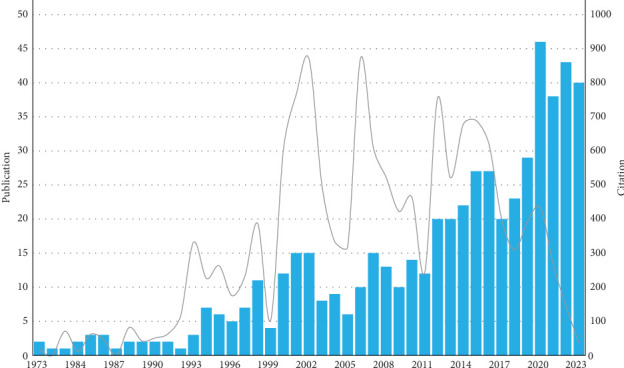
Analysis of global research trend of clinical trials on the treatment of temporomandibular disorders from 1973 to 2023.

The bibliometric analysis included a total of 559 CTs, according to the flow diagram (Figure 1). As shown in Figure 2, since the 2000s there has been a trend towards an increase in CTs on TMD treatments, being more notable between the years 2012–2023 with an average number of 29.6 publications in this period. The year with the greatest number of publications was 2020 (*n* = 46). The number of times CTs were cited per year was variable, with the maximum peaks being found within three distinct periods; between 1973–1999 (average 35.1), 2000–2011 (average 46.9), and 2012–2023 (average 14.8). The highest numbers of citations for each period were observed in 1998 (387 citations), 2006 (871 citations), and 2012 (755 citations), respectively (Figure 2).

### 3.2. Characteristics of CTs (Supporting Information)

In total, 66.9% of the CTs applied only conservative therapy (COT) modalities (*n* = 374) and 20.6% only minimally invasive therapies (MITs) (*n* = 115) (Table S1). COTs were used in the earliest CTs (1973), while open joint surgery (OJS) was evaluated after ~1998 (Figure S1). The most commonly used COTs were physiotherapy (134/559) and occlusal splinting (126/559) (Table S2). The documented use of nonsteroidal analgesic drugs, anxiolytics, or antidepressants in CTs dates back to 1973, being the earliest therapies used to treat TMD, followed by physiotherapy (1983) and occlusal splinting (1983) (Figure S2). MITs, such as intra-articular injections (135/559) and arthrocentesis (59/559), were the most studied in CTs (Table S2). The use of intra-articular injections to treat TMJ disorders dates back to about 1984, and we found that CTs evaluated intra-articular injection with botox, hyaluronic acid, platelet-rich plasma, and ozone (Figure S3). In 17.7% of the CT combined therapies were studied; that is, the joint administration of two or more therapies was analyzed; 89.9% evaluated treatments for painful symptoms and diagnostic modalities were related to TMJ in 78.7%. Among the articles found, 79.8% were randomized controlled trials. There was a lower frequency of uncontrolled trials (12.2%) and most studies (66.5%) used a control group other than placebo (Table S1).

### 3.3. Analysis of the Cooperative Relationship

#### 3.3.1. Countries

**Figure 3:**
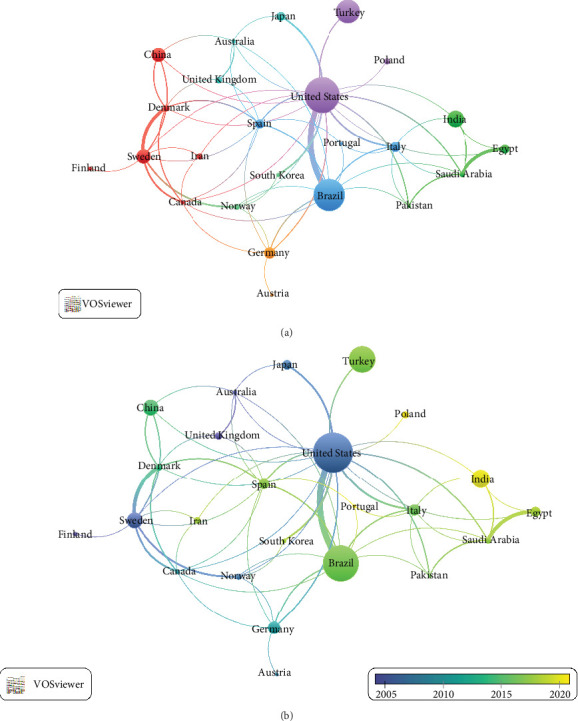
(A) Cluster graph of countries publishing clinical trials on the treatment of temporomandibular disorders. (B) Cluster graphs of countries with timeline view.

Approximately 51 countries in the world have published at least one CT on TMD treatment.  Table 1 shows the 10 countries with the highest number of published CT on TMD treatment, which accounted for 75.5% (*n* = 422) of the total. The United States (US) had the highest number of CTs (*n* = 83) followed by Brazil (*n* = 79) and the US is the country with the highest impact, with an average number of citations of 47.5 citations per CT. In addition, 25 countries published at least five studies involving collaboration with another country (Figure 3A), with the main collaborations being between the US (*n* = 83) and Brazil (*n* = 79), Sweden (*n* = 33) and Denmark (*n* = 12), and Egypt (*n* = 21) and Saudi Arabia (*n* = 12). Figure 3B shows that the US, Sweden, and the United Kingdom were the first to perform CTs, while India and Poland were the last.

#### 3.3.2. Institutions

**Figure 4:**
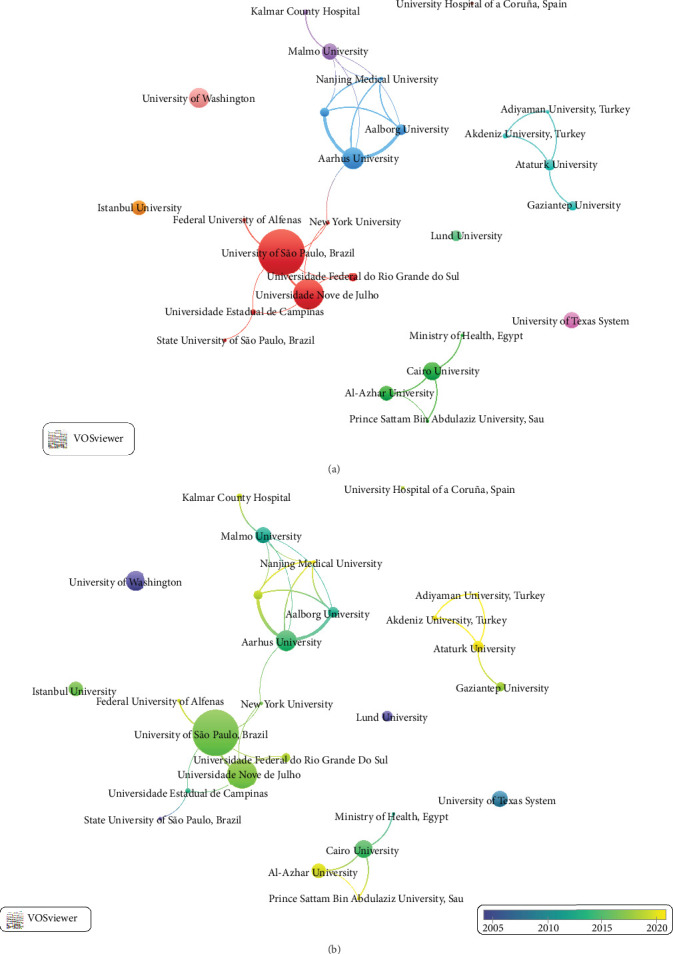
(A) Clustering graph of institutions. (B) Cluster graph of institutions with timeline view.

From 1973–2023 ~692 institutions have published CTs.  Table 2 shows the top 10 institutions with the highest scientific production in relation to CTs on TMD, with the University of São Paulo contributing the largest number (*n* = 28), followed by the Universidade Nove de Julho (*n* = 17). The institution with the highest impact was the University of Washington (*n* = 13) with an average of 91.1 citations. The strongest collaborations were found between Aarhus University and Aalborg University, as well as between the University of São Paulo and Universidade Nove de Julho (Figure 4A). Institutions such as the University of Washington and Lund University were the first to perform CTs on TMD treatment (Figure 4B).

#### 3.3.3. Authors

**Figure 5:**
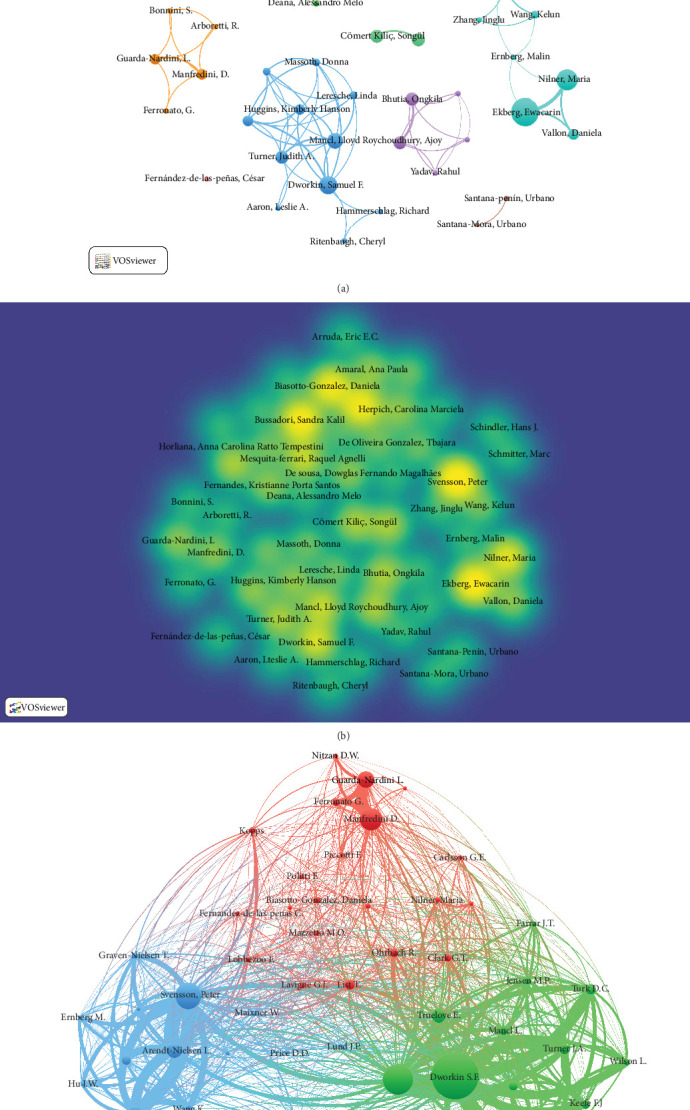
(A) Cluster graph of authors. (B) Cluster graph of authors with heatmaps of terms. (C) Co-citation analysis of authors.

We identified 2260 authors who published CTs and the top 10 most productive authors were Peter Svensson (*n* = 12), EwaCarin Ekberg (*n* = 11) and Sandra K. Bussadori (*n* = 11), from the University of Aarhus, Lund University, and Universidade Nove de Julho, respectively. However, Judith A. Turner (*n* = 7) and Samuel F. Dworkin (*n* = 9) were the authors with the highest impact according to the average number of citations (Table 3). The collaborative network in Figure 5A,B shows Peter Svensson, Sandra K. Bussadori, Samuel F. Dworkin, and EwaCarin Ekberg as the central hubs of their clusters. Strong collaboration was also found between the conglomerates of Sandra K. Bussadori and Daniela A. Biasotto-Gonzalez. Likewise, Samuel F. Dworkin and EwaCarin Ekberg are the original authors in relation to these studies. On the other hand, Samuel F. Dworkin and Linda LeResche performed CTs on similar therapies (Figure 5C).

#### 3.3.4. Journals

**Figure 6:**
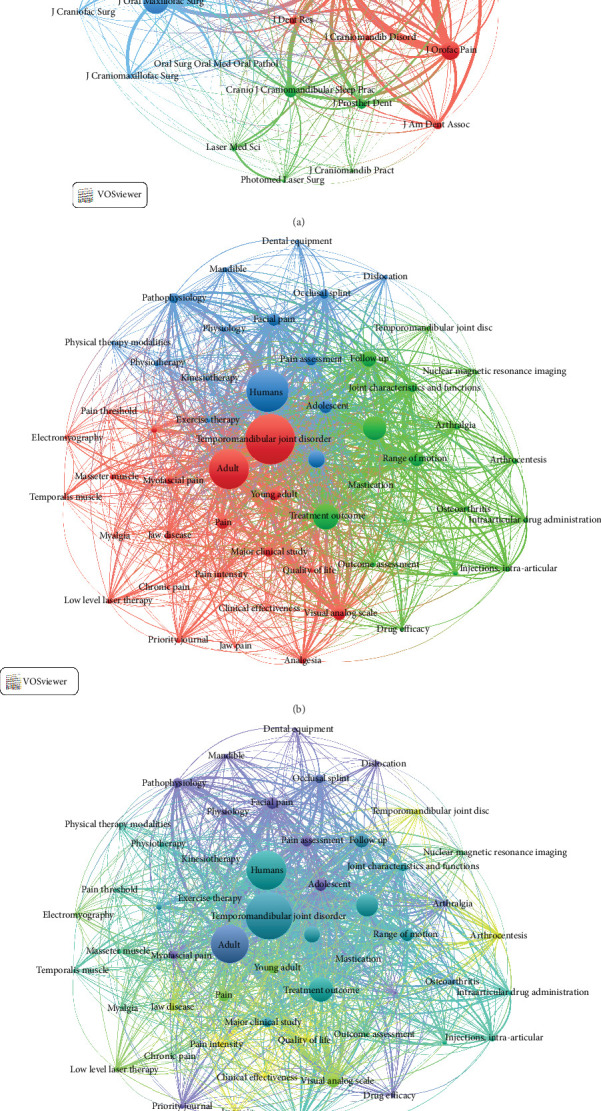
(A) Co-citation analysis of journals. (B) Cluster graph of keywords. (C) Cluster graph of keywords with timeline view.

There were 198 journals that published CTs related to TMD treatments. Each of the journals belonging to the top 10 have published at least 11 articles and represent 38.5% (*n* = 215) of the total number of articles analyzed. The Journal of Oral and Maxillofacial Surgery (*n* = 39) and Cranio—Journal of Craniomandibular and Sleep Practice (*n* = 33) were the journals that have published the most CTs, and the most influential was the Journal of Oral Rehabilitation with an average number of citations of 3.6 (Table 4).  Figure 6A shows the main co-citations; first, between the Journal of Oral and the Maxillofacial Surgery and Oral Surgery, Oral Medicine, Oral Pathology and Oral Radiology and, second, between the Journal of Oral & Facial Pain and Headache and the Journal of Oral & Facial Pain and Headache. These co-citations are probably due to the fact that they present similar topics in terms of the TMD treatments evaluated.

#### 3.3.5. Most Cited CTs

The most cited articles were “Short- and long-term efficacy of brief cognitive-behavioral therapy for patients with chronic temporomandibular disorder pain: A randomized, controlled trial” by Turner et al. [18] published in 2006 by Pain, followed by “A randomized clinical trial using research diagnostic criteria for TMDs-axis II to target clinic cases for a tailored self-care TMD treatment program” and “A randomized clinical trial of a tailored comprehensive care treatment program for temporomandibular disorders” by Dworkin et al. [19] published in 2022 by the Journal of Orofacial Pain. The study by Turner et al. [18] evaluated the efficacy of a cognitive-behavioral therapy for chronic pain in TMD in a CT. The most cited studies by Dworkin et al. [19] compared COTs such as therapies focused on psychosocial disorders and conventional occlusal therapies (Table 5).

#### 3.3.6. Analysis of Keywords

**Figure 7:**
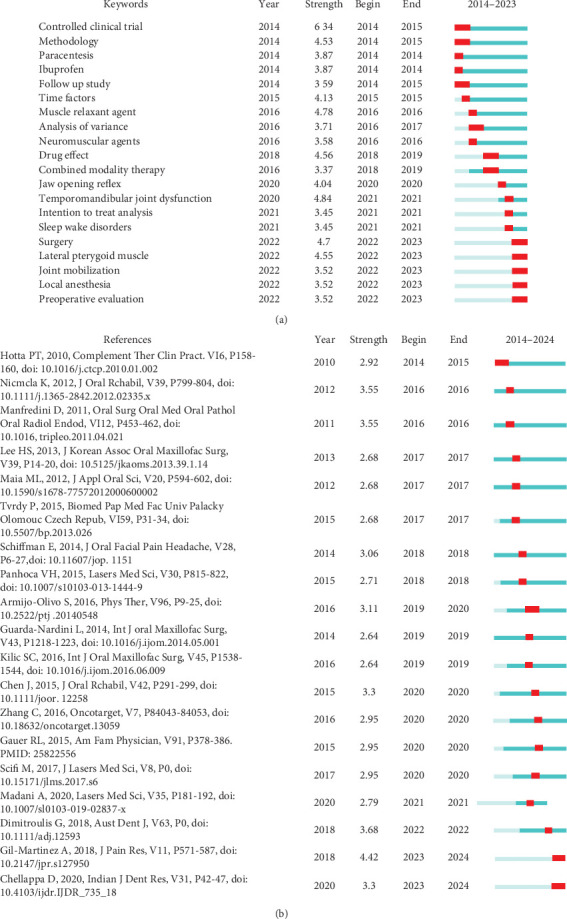
(A) Top 20 keywords with the strongest citation bursts. (B) References with the strongest burst.

A total of 2644 keywords were found, and the co-occurrence analysis (Figure 6B) shows those repeated at least 29 times and three clusters can be distinguished. The first (red) is related to treatments at the level of the masticatory muscles, the second (green) is related to treatments at the level of the TMJ and the third (blue) is related to treatments related to pain. A dynamic network is evident, showing that muscle and joint therapies were applied earlier, while painful pathologies have been addressed in recent years. According to the burst analysis (Figure 7A), 20 keywords are distinguished from the rest by their usage. Between 2014 and 2016, “controlled clinical trial” and “muscle relaxant agent” were the most used, while between 2018 and 2021, “Temporomandibular Joint Dysfunction” and “Drug effect” were the most relevant, and between 2022 and 2023, “Surgery” and “Lateral Pterygoid Muscle” were the most applied in research. This indicates that treatments for TMJ and masticatory muscles in individuals with TMD are gaining interest with their use being more frequently evaluated in controlled trials since 2014.

#### 3.3.7. Analysis of References

Figure 7B shows the top 20 most cited references and those of greatest interest in the majority of CT. The first is that of Gil-Martinez et al. [20] entitled “Management of pain in patients with temporomandibular disorder (TMD): challenges and solutions,” which proposed a biobehavioral model (physiotherapy, psychological, and dental therapy) as a clinical solution for patients with craniofacial pain and TMD. The second is the study by Dimitroulis et al. [21] entitled “Management of temporomandibular joint disorders: A surgeon's perspective,” which analyzes the treatment possibilities for TMD and proposes multidisciplinary methods that are adjusted according to the patients' needs.

## 4. Discussion

The results of this bibliometric analysis show that between 1973 and 2023 there has been a considerable increase in the publication of treatment-related CTs for TMD, albeit with some irregularities in relation to the types of treatment used. The most prolific countries were the US and Brazil, explained by international cooperation and establishing marked gaps with countries such as Spain and Egypt. Most CTs were controlled and randomized and evaluated mainly COTs such as physiotherapy, followed by occlusal splints as the dental treatment of choice and phototherapy. MITs such as intra-articular injections played an important role during the treatment of patients with TMD. The University of São Paulo and Universidade Nove de Julho, both in Brazil, were the most important institutions in scientific production, while the University of Washington had the greatest impact. Peter Svensson was the most prolific author, while Judith A. Turner was the most influential. The Journal of Oral and Maxillofacial Surgery contributed the most to knowledge about TMD treatment, but Oral Surgery, Oral Medicine, Oral Pathology, and Oral Radiology had the publications with the greatest impact on the scientific community.

The bibliometric studies published to date have explored the scientific production on the diagnosis, treatment, prevention, and classification of TMD in a general way [22–25], while others have done so in a more specific way [26, 27], However, no study of this type has analyzed CTs, which evaluate the effectiveness of treatments in a more reliable way and, as the design is more scientifically sound, imply greater rigor on the part of the authors and expert reviewers of these studies. In the present analysis, the main authors considered authorities in the execution of CTs in TMD treatment showed that they work in very delimited clusters, which means that they need to expand their international collaboration networks for better understanding and the application of these designs in different populations and healthcare systems.

Within the keyword co-occurrence analysis, three main research clusters (masticatory muscle treatment, TMJ, and pain) were distinguished, with muscle and joint therapies being applied earlier compared to pain-related therapies. In addition, randomized controlled CTs have gained greater prominence in research since 2014, indicating that pain treatments in TMD are gaining interest with more rigorous research methodologies. We found that since 1973 COTs began to be evaluated in CTs on the treatment of musculoskeletal pain and, thereafter, multiple treatments have been tested due to their high efficacy, which is concordant with current reviews [28]. Physiotherapy, in conjunction or not with other therapies, has achieved excellent effects which have been demonstrated in multiple studies on both the recovery of function, decrease of inflammation, and reduction of pain and other TMD symptoms [7, 29–32]. However, it is still necessary to strengthen research with more rigorous CTs justified by the new treatment strategies currently being promoted [21, 33], which probably explain the increase in the production of CTs in recent years.

On the other hand, intra-articular injections began to gain prominence in CTs performed between 1983 and 1988, with the aim of improving the treatment that COTs cannot resolve, such as TMJ and capsule inflammations associated with pain. A range of therapies were found in CTs on different injectable substances at the joint level, such as corticosteroids [34–37], solutions with dextrose [38, 39], hyaluronic acid [40, 41], morphine [42, 43], platelet-rich plasma [44, 45], botulinum toxin [46], or ozone gas [47], among others. However, the effectiveness of most of these treatments remains to be demonstrated in CTs. Likewise, research in this aspect is somewhat scarce and shows a tendency to increase at a slow pace. It is expected that in the future more CTs on treatments, such as botulinum toxin and ozone gas at the TMJ level, will be carried out. Likewise, OJS, such as arthroplasty, represent a small group of therapies that are available for multiple cases and have demonstrated efficacy in improving joint mobility and function [48, 49].

This bibliometric study showed that the most cited CTs on TMD treatment [18, 50] have been largely associated with patients with psychosocial disturbances, suggesting that cognitive therapies in conjunction with COTs may be effective in these cases and should be applied jointly in daily clinical practice. This has reinforced the old paradigm suggesting that psychosocial disorders are the main perpetuating factors of chronic TMD symptoms. Alongside this paradigm, another that has been controversial over the years is the association between TMD and occlusal aspects. In this regard, the CTs analyzed showed a reduction in production for therapies applying occlusal splints since 2014. Although CTs evaluating occlusal adjustment or occlusal therapy as a solution for TMD symptoms have shown a trend to an increase, the number of CTs in this regard remains low. However, the number of CTs to elucidate the role of occlusion in the origin of TMD symptoms is still considerable and and more will likely appear in the future.

The most widely cited references corroborate that CTs are oriented toward further research in COTs [51] and neglect occlusal treatments [52]. The majority of CT analyzed in this study used diagnostic methods such as Diagnostic Criteria for TMDs (DC/TMD) to classify TMD patients, which explains why the article by Shiffman et al. [53]. “Diagnostic Criteria for Temporomandibular Disorders (DC/TMD) for Clinical and Research Applications: recommendations of the International RDC/TMD Consortium Network*⁣*^*∗*^ and Orofacial Pain Special Interest Group†” and the article by Manfredini et al. [54] “Research diagnostic criteria for TMDs: a systematic review of axis I epidemiologic findings” has been important TMD diagnosis-related references in the burst analysis. It would be relevant to determine whether trends in the use of diagnostic methods for TMD are undergoing changes in order to clarify the comparability of the results found in CTs.

The limitations of this study may be related to the search for information. New studies not covered by the time period analyzed may have appeared to date. In addition, the PubMed database was not explored, and thus, there is a possibility of not having used all the available evidence. However, we used powerful databases, such as Scopus and WoS, in which the highest quality research is found. There is a probable information bias in the reporting of citations by not eliminating self-citations, due to the limitations of CiteSpace and VOSviewer, which would lead to an overestimation of the impact or a misconception of centrality in the networks. However, the findings are considered to be a good approximation of what has historically occurred with quality scientific production regarding CTs on TMD. Future CTs on TMD treatments should focus on generating more evidence on the effectiveness of emerging COTs and MITs due to their wide variety and the combinations that can be applied taking into account a pathological classification system. Likewise, it is important that the results of this analysis allow the strengthening of collaboration in the production of CTs through multicenter consortiums led by the institutions with the greatest impact, raising methodological rigor and incorporating emerging technologies in the treatment of TMD.

## 5. Conclusion

Research through CTs evaluating treatments for TMD over the last 50 years peaked in 2020, indicating the need for further studies on conservative and MITs in this disorder. Research in new therapies evidenced an irregular publication pattern with an upward trend. This bibliometric analysis demonstrates the flexibility of CTs applied in the study of TMD with a great contribution in the knowledge of this disorder by US and Brazil demonstrating notable differences in comparison with other contributing countries. Treatments applied to treat painful symptomatology at the muscular level occupy an important role in this analysis, after symptomatology at the TMJ level. The analysis of the thematic trends showed small changes in the new treatment approaches for TMD symptomatology, likely indicating that the use of COTs, such as phototherapy or electrostimulation, or MITs, such as joint injection with dextrose, hyaluronic acid, morphine, platelet-rich plasma, or ozone, among others, will appear in the near future. In addition, we found a paradigm shift in treatment strategies from purely occlusal to biobehavioral.

## Figures and Tables

**Table 1:** Top 10 countries with most relevant publications on the treatment of temporomandibular disorders.

Rank	Country	Documents	Citations	Average citations	Leading contributors (number of publications)
1	United States	83	3945	47.5	Dworkin, Samuel F. (9); Mancl, Lloyd (7)
2	Brazil	79	1750	22.2	Ekberg, EwaCarin (11); Bussadori, Sandra Kalil (11)
3	Turkey	58	1350	23.3	Cömert Kiliç, Songül (6); Güngörmüş, Metin (5)
4	India	42	303	6.7	Bhutia, Ongkila (5); Roychoudhury, Ajoy (5)
5	China	34	325	9.6	Wang, Kelun (3)
6	Sweden	33	1147	34.8	Svensson, Peter (12); Biasotto-Gonzalez, Daniela A. (9)
7	Italy	26	768	29.5	Guarda-Nardini, Luca (6); Manfredini, Daniele (5)
8	Germany	23	376	16.3	Schmitter, Marc (2)
9	Spain	23	530	23.0	Fernández-de-Las-Peñas, César (3)
10	Egypt	21	314	15.0	Hegab, Ayman F. (4)

**Table 2:** Top 10 institutions publishing clinical trials on the treatment of on the treatment of temporomandibular disorders.

Rank	Institution	Documents	Citations	Average citations	Countries
1	University of São Paulo	28	836	29.9	Brazil
2	Universidade Nove de Julho	17	319	18.8	Brazil
3	University of Washington	13	1184	91.1	United States
4	Malmö University	13	261	20.1	Sweden
5	Aarhus University	12	254	21,2	Denmark
6	Cairo University	11	737	61.4	Egypt
7	University of Texas System	10	445	44.5	United States
8	Al-Azhar University	8	151	18.9	Egypt
9	Istanbul University	8	65	8.1	Turkey
10	Ataturk University	7	115	16.4	India

**Table 3:** Top 10 authors publishing clinical trials on the treatment of temporomandibular disorder.

Rank	Author	Documents	Citations	Average publication year	Average citation	Affiliated institution	Country
1	Svensson, Peter	12	254	2014	21.2	University of Aarhus	Denmark
2	Ekberg, EwaCarin	11	442	2002	40.2	Lund University	Sweden
3	Bussadori, Sandra Kalil	11	166	2016	15.1	Universidade Nove de Julho	Brazil
4	Biasotto-Gonzalez, Daniela A.	10	230	2015	23	Universidade Nove de Julho	Brazil
5	Politti, Fabiano	9	220	2016	22	Universidade Nove de Julho	Brazil
6	Nilner, Maria	9	463	2001	51.4	Malmö University	Sweden
7	Dworkin, Samuel F.	9	849	2002	94.3	University of Washington	United States
8	Mesquita-Ferrari, Raquel Agnelli	7	101	2018	14.4	Universidade Nove de Julho	Brazil
9	Turner, Judith A.	7	898	2002	128.3	University of Washington	United States
10	Fernandes, Kristianne Porta Santos	7	86	2018	12.3	Universidade Nove de Julho	Brazil

**Table 4:** Top 10 journals with most relevant publications on the treatment of TMD.

Rank	Journal	Documents	Citations	Average citations	Impact factor (2023)	Average publication year
1	Journal of oral and maxillofacial surgery	39	1108	28.4	1.9	2013
2	Cranio - journal of craniomandibular and sleep practice	33	762	23.1	1.6	2008
3	Journal of oral rehabilitation	29	989	34.1	3.6	2013
4	International journal of oral and maxillofacial surgery	23	649	28.2	2.8	2014
5	Journal of cranio-maxillofacial surgery	20	396	19.8	1.3	2018
6	Journal of oral & facial pain and headache	20	358	32.5	2.4	2014
7	Acta odontologica scandinavica	17	642	37.7	2.0	2006
8	Oral surgery, oral medicine, oral pathology and oral radiology	16	716	44.8	2.3	2003
9	British journal of oral and maxillofacial surgery	15	484	32.3	1.8	2007
10	Journal of craniofacial surgery	12	186	15.5	1.2	2015

**Table 5:** Top 10 publications with the most citations on the treatment of temporomandibular joint disorders.

Rank	Title	Source journal	Citations	Citation density	Publication year
1	Short- and long-term efficacy of brief cognitive-behavioral therapy for patients with chronic temporomandibular disorder pain: A randomized, controlled trial	Pain	216	12.7	2006
2	A randomized clinical trial using research diagnostic criteria for temporomandibular disorders-axis II to target clinic cases for a tailored self-care TMD treatment program	Journal of orofacial pain	203	9.7	2002
3	A randomized clinical trial of a tailored comprehensive care treatment program for temporomandibular disorders	Journal of orofacial pain	173	8.2	2002
4	Effects of intraoral appliance and biofeedback/stress management alone and in combination in treating pain and depression in patients with temporomandibular disorders	The journal of prosthetic dentistry	146	4.9	1993
5	The efficacy of traditional, low-cost and nonsplint therapies for temporomandibular disorder: A randomized controlled trial	Journal of the American dental association	143	8.4	2006
6	Effectiveness of low-level laser therapy in temporomandibular disorder	Scandinavian journal of rheumatology	139	6.9	2003
7	Biomet microfixation temporomandibular joint replacement system: a 3-year follow-up study of patients treated during 1995 to 2005	Journal of oral and maxillofacial surgery	136	12.4	2012
8	Brief group cognitive-behavioral intervention for temporomandibular disorders	Pain	132	4.5	1994
9	Effectiveness of low-level laser therapy in temporomandibular joint disorders: a placebo-controlled study.	Photomedicine and laser surgery	132	8.3	2007
10	Adrenergic dysregulation and pain with and without acute beta-blockade in women with fibromyalgia and temporomandibular disorder	Journal of pain	129	9.2	2009

## Data Availability

The data supporting the findings of this study will be accessible by contacting the corresponding author. The same will be available by making a request to the author Yens Valerio Mendoza Martiarena, email: ymendoza@cientifica.edu.pe.

## Supporting Information

Supporting Information The Supporting Information describes the characteristics of the clinical trials analyzed (Table S1), the types of treatment (Table S2), the trends from 1973 to 2023 of the treatment modalities used in the studies (Figure S1), the trends from 1973 to 2023 of the use of conservative (Figure S2) and minimally invasive therapies (Figure S3).
