# Supplementary material for: Bibliometric Analysis of Research Trends Related to the Publication of Clinical Trials on the Treatment of Temporomandibular Disorders Between 1973 and 2023
Source: Int J Dent. 2025 Aug 6;2025:8594730. doi: 10.1155/ijod/8594730 (PMC12350018; doi:10.1155/ijod/8594730)
Supplement: Supporting Information — The Supporting Information describes the characteristics of the clinical trials analyzed (Table S1), the types of treatment (Table S2), the trends from 1973 to 2023 of the treatment modalities used in the studies (Figure S1), the trends from 1973 to 2023 of the use of conservative (Figure S2) and minimally invasive therapies (Figure S3). [file 8594730.f1.docx]

**Supplementary material**

In total, 66.9% of the CTs applied only conservative therapy modalities (COTs) (n = 374) and 20.6% only minimally invasive therapies (MITs) (n = 115) (Table S1). COTs were used in the earliest CTs (1973), while open joint surgery (OJS) was evaluated after approximately 1998 (Figure S1). The most commonly used COTs were physiotherapy (134/559) and occlusal splinting (126/559) (Table S2). The documented use of non-steroidal analgesic drugs, anxiolytics or antidepressants in CTs dates back to 1973, being the earliest therapies used to treat TMD, followed by physiotherapy (1983) and occlusal splinting (1983) (Figure S2). MITs, such as intra-articular injections (135/559) and arthrocentesis (59/559), were the most studied in CTs (Table S2). The use of intra-articular injections to treat TMJ disorders dates back to about 1984, and we found that CTs evaluated intra-articular injection with botox, hyaluronic acid, platelet-rich plasma, and ozone (Figure S3). In 17.7% of the CT combined therapies were studied; that is, the joint administration of two or more therapies were analyzed; 89.9 % evaluated treatments for painful symptoms and diagnostic modalities were related to TMJ in 78.7 %. Among the articles found, 79.8% were randomized controlled trials. There was a lower frequency of uncontrolled trials (12.2%) and most studies (66.5%) used a control group other than placebo (Table S1).

Table S1. Characteristics of the clinical trials analyzed (n = 559).

| Characteristics | | n | % |
| --- | --- | --- | --- |
| Treatment Modalities | |  |  |
|  | COT | 374 | 66.9 |
|  | MIT | 115 | 20.6 |
|  | COT,MIT | 38 | 6.8 |
|  | OJS | 22 | 3.9 |
|  | MIT,OJS | 5 | 0.9 |
|  | COT,MIT,OJS | 3 | 0.6 |
|  | COT, OJS | 2 | 0.3 |
| Diagnoses Modalities | |  |  |
|  | TMJ,M | 270 | 48.3 |
|  | TMJ | 167 | 29.9 |
|  | M | 119 | 21.3 |
|  | TMJ,M,HA | 2 | 0.4 |
|  | TMJ,HA | 1 | 0.2 |
| Common Diagnoses | |  |  |
|  | PC | 440 | 78.7 |
|  | PC,NPC | 62 | 11.1 |
|  | NPC | 57 | 10.2 |
| Combined therapy | |  |  |
|  | No | 460 | 82.3 |
|  | Yes | 99 | 17.7 |
| Placebo | |  |  |
|  | No | 372 | 66.5 |
|  | Yes | 119 | 21.3 |
| Experimental_study | |  |  |
|  | Randomized controlled trial | 446 | 79.8 |
|  | Uncontrolled trial | 68 | 12.2 |
|  | Non-randomized controlled trial | 45 | 8.1 |

COT, conservative therapy; MIT,minimally invasive therapy; OJS, open surgery; TMJ, temporomandibular joint; M, muscles; HA, Headache; PC, pain condition; NPC, non pain condition

Table S2. Treatment type

| Treatment Modalities | Treatment type | n┼ |
| --- | --- | --- |
| COT | Physiotherapy | 134 |
| COT | Occlusal splint | 126 |
| COT | Phototherapy: low-level laser therapy (LLLT) and light-emitting diode (LED) therapy | 66 |
| COT | Analgesics, non-steroidal anti-inflammatory drugs (NSAIDs), anxiolytics, and anti-depressants | 61 |
| MIT | Simple and double-puncture arthrocentesis | 59 |
| MIT | Hyaluronic acid intra-articular injection | 45 |
| COT | Psychosocial therapy | 40 |
| MIT | Drugs intra-articular injection | 31 |
| COT | Dry-needling, acupuncture | 31 |
| MIT | Platelet-rich plasma intra-articular injection | 27 |
| MIT | Arthroscopic surgery | 22 |
| COT | Electrical stimulation | 19 |
| COT | Occlusal adjustment / occlusal therapy | 17 |
| MIT | Botulinum toxin intra-articular injections | 15 |
| COT | Diet modification | 11 |
| OJS | Arthroplasty | 11 |
| COT | Ultrasound | 9 |
| MIT | Solutions intra-articular injection | 8 |
| COT | Extracorporeal shock wave therapy | 6 |
| OJS | Open Joint Surgery | 5 |
| COT | Stress reduction techniques | 4 |
| COT | Naturopathic medicine | 3 |
| OJS | Eminoplasty/eminectomy | 3 |
| OJS | Condylar discopexy | 3 |
| MIT | Analgesics intra-articular injection | 2 |
| MIT | Autologous blood intra-articular injection | 2 |
| COT | Magnetostimulation, magnetoledotherapy, magnetolaserotherapy | 2 |
| OJS | Orthognathic surgery | 2 |
| OJS | Subfascial approach | 2 |
| OJS | Osteotomy | 2 |
| MIT | Ozone gas intra-articular Injections | 2 |
| OJS | Mandibular fixation | 1 |
| OJS | Osteoarthrectomy | 1 |
| OJS | Stock alloplastic | 1 |
| OJS | Surgical reconstruction | 1 |
| MIT | Adipose tissue intra-articular injection | 1 |
| COT | Cryotherapy | 1 |
| MIT | Human placental extract intra-articular injection | 1 |
| MIT | Nucleated cell (BMNc) intra-articular injection | 1 |
| COT | Traditional medicine | 1 |

┼, the absolute frequencies do not add up to 559. COT, conservative therapy; MIT,minimally invasive therapy; OJS, open surgery

**Figure S1.** Trend of treatment modalities evaluated in clinical trials on temporomandibular disorder.

**Figure S2.** Trend of the main conservative therapies evaluated in clinical trials on temporomandibular disorders.

**Figura S3.** Trend of the main minimally invasive therapies evaluated in clinical trials in temporomandibular disorder.
